# Occurrence of the family *Enterobacteriaceae* (sensu stricto) in cow’s milk and their antimicrobial resistance

**DOI:** 10.5455/javar.2026.m1019

**Published:** 2026-03-10

**Authors:** Zuzana Hanzelová, Eva Dudriková, František Zigo, Viera Lovayová

**Affiliations:** 1 Center of Clinical and Preclinical Research MEDIPARK, Faculty of Medicine, Pavol Jozef Šafárik University in Košice, Trieda SNP 1, 040 11 Košice, Slovakia; 2 Department of Food Hygiene, Technology and Safety, University of Veterinary Medicine and Pharmacy, Komenského 73, 041 81 Košice, Slovakia; 3 Department of Animal Nutrition and Husbandry, University of Veterinary Medicine and Pharmacy, Komenského 73, 041 81 Košice, Slovakia; 4 Department of Medical and Clinical Microbiology, Faculty of Medicine, Pavol Jozef Šafárik University in Košice, Trieda SNP 1, 040 11 Košice, Slovakia

**Keywords:** *Enterobacteriaceae* (sensu stricto), pasteurization, resistance genes

## Abstract

**Objectives:** The *Enterobacteriaceae* family is a group of bacteria that serve as indicators of milk collection hygiene at the farm level. The objective of this study was to detect and identify members of this family in raw cow’s milk and pasteurized milk and to evaluate their antimicrobial susceptibility and the presence of resistance genes.

**Materials and Methods:** A total of 14 samples of raw bulk milk and 14 samples of pasteurized milk were collected from different dairy plants in Slovakia. Isolates were identified at the family level by PCR and at the species level by MALDI-TOF MS. Ten antibiotics (ampicillin, cefoxitin, ceftizoxime, cefazolin, kanamycin, streptomycin, tetracycline, gentamicin, ofloxacin, and chloramphenicol) were tested using the disc diffusion method. Genes that confer antimicrobial resistance to beta-lactam antibiotics were detected by PCR using gene-specific primers.

**Results:** *Klebsiella oxytoca* was the most frequently identified isolate (30.0%), followed by *Enterobacter cloacae* (13.3%) and *Citrobacter gillenii* (13.3%). According to the CLSI criteria, 22 of the 30 isolates (73.3%) were classified as cefoxitin-resistant, whereas EUCAST classified 26/30 (86.7%) as cefoxitin-resistant. The presence of resistance genes (at least one of the genes tested) was confirmed in 26 isolates. In 20 isolates, *bla*_TEM_ was found; in 11, *bla*_CTX–M_; and in 10, *ampC* genes.

**Conclusions:** Our findings highlight the absence of *Enterobacteriaceae* in pasteurized milk, discrepancies in evaluation between the CLSI and EUCAST systems, a high level of beta-lactam resistance, and the presence of resistance genes detected only in raw milk samples.

## 1. Introduction

The family *Enterobacteriaceae* belongs phylogenetically to the order *Enterobacterales*, the class *Gammaproteobacteria*, and the phylum *Proteobacteria*. This group of bacteria was created in 1937 and was initially based on representatives of the genus *Enterobacter. Enterobacteriaceae* have undergone several major taxonomic revisions. A significant change occurred when some genera originally classified within *Enterobacteriaceae* were reassigned to separate families. These changes were driven by new data obtained through gene sequencing technologies [1, 2].

In animals, economically important *Enterobacteriaceae* infections include gastrointestinal tract diseases. The most common causes of mastitis in cattle are *Citrobacter koseri, Enterobacter aerogenes*, and *Klebsiella pneumoniae*, belonging to this family. In humans, they are responsible for more than 50% of nosocomial infections. They are responsible for almost 50% of septicemias, 60–70% of intestinal infections, and 70% of urinary tract infections [3, 4].

Milk is a suitable medium for the growth of various microorganisms because it is rich in nutrients, has a high-water content, and maintains a neutral pH. Rapid proliferation of microorganisms, particularly at high ambient temperatures, can alter the physical and chemical composition of milk. As summarized by Chege and Ndungu [5], raw milk can become contaminated at various points along the processing chain and from multiple sources. In general, microorganisms can contaminate raw milk in two primary ways. The first is endogenous contamination, where milk is contaminated via direct transmission from a sick animal (e.g., systemic infections or mastitis). The second is exogenous contamination, which occurs during or after milking through contact with feces, the external parts of the udder, teats, the environment, or equipment [6].

The genera most isolated from the *Enterobacteriaceae* family in milk and dairy products include *Citrobacter, Enterobacter, Escherichia, Klebsiella*, and *Kluyvera*. The genus *Enterobacter* is the most predominant in raw and pasteurized milk. Species of the genus *Escherichia* are found in approximately 10–17% of raw milk samples, and in less than 10% of all coliforms in pasteurized milk [7, 8, 9]. The presence of *Enterobacteriaceae* in raw milk is a reason to use effective heat treatment, as required by current legislation. In accordance with EU Regulation 2073/2005, the acceptance criterion for pasteurized milk is set at m = M = 10 CFU/ml with an allowable number of exceeding samples (c) of zero. Consequently, any detection below this 10 CFU/ml threshold is classified as legally satisfactory, indicating that the manufacturing process is operating within controlled hygiene parameters [10]. Pasteurization should eliminate *Enterobacteriaceae* in milk for further manufacturing. This is why some studies focus on undesirable bacteria in milk after heat treatment, as their presence after pasteurization may affect, among other things, subsequent processing.

As is well known, antimicrobial resistance (AMR) in *Enterobacteriaceae* from raw materials of animal origin may be closely related to the use of antibiotics in therapeutic interventions on farms, particularly for the treatment of mastitis in dairy cows. The AMR situation shows specific characteristics depending on therapeutic practices in a given country. This applies to both human and animal populations and their interconnectedness. [11, 12].

The application of antibiotics can affect the food industry, as antibiotic resistance in animals can be transferred to food products. There is also an indirect risk of horizontal transfer of resistance genes to pathogenic microorganisms at different points in the food chain [13]. Multi-resistance is often associated with the presence of *Escherichia coli* and *Salmonella* spp., which are considered the most common foodborne pathogens. According to Fakruddin et al. [14], multi-resistant *K. pneumoniae* bacteria were detected in raw milk samples, and multi-resistant bacteria of the genera *Enterobacter, Citrobacter*, and *Klebsiella* were also detected in various food samples, including powdered milk. Currently, the positions on the assessment of antibiotic susceptibility across the two systems, namely CLSI and EUCAST, are a subject of debate due to many differences observed when both are used in parallel. AMR is a global problem, and unifying both systems would be appropriate.

Many researchers have highlighted the potential risk of transmission of ESBL (extended-spectrum beta-lactamases), *ampC* (aminopenicillin-inactivating cephalosporinases), and CP (carbapenemases) produced by bacteria of the *Enterobacteriaceae* family to consumers through milk and dairy products sold directly from farms, such as *E. coli* and *K. pneumoniae*, that confer resistance to many commonly used antibiotics [15, 16]. Genes encoding ESBLs are most frequently found in transposons or plasmid insertion sequences. Consequently, animal production has become a major concern in recent decades due to its close connection with the food chain and its potential role as a reservoir, vehicle, or transmission route for the dissemination of ESBLs. In the past, these genes were mainly investigated in isolates exhibiting phenotypic resistance. However, it was later discovered that their presence could also be detected in isolates that were not resistant [17].

Based on the above, the aim of our study was to determine the occurrence of *Enterobacteriaceae* in raw bulk milk and milk after pasteurization, identify them, assess their antimicrobial resistance profiles and the presence of resistance genes, and determine the occurrence of *Enterobacteriaceae* in raw bulk milk and milk after pasteurization. Isolates were identified and classified according to current taxonomic systems. Additionally, evaluating the two standard systems for assessing AMR may reveal differences between them. The findings would contribute to a better understanding of AMR in foodborne bacteria and underscore important implications for food safety and public health.

## 2. Materials and Methods

### 2.1. Ethical approval

Ethical approval was not required for this study because it involved the microbiological analysis of milk samples collected from dairy plants and did not involve human participants or experimental animals.

### 2.2. Isolation and identification of Enterobacteriaceae

A total of 14 samples of raw bulk milk and 14 samples of pasteurized milk were collected from four dairy plants (west, north, east, and southeast) in Slovakia, which are focused on artisanal cheesemaking. The samples were taken in accordance with ISO 7218 [18]. Dairies receive milk daily from farms in close proximity; the dairy in the southeast also has its own farm with dairy cows on-site. Our visits in diaries were announced in advance to each dairy plant. The samples of pasteurized milk were taken after heat treatment of each raw bulk milk; they matched each other. Each sample (raw or pasteurized milk), with a volume of 500 ml, was transported at 4°C without the addition of preservatives to the laboratory at the University of Veterinary Medicine and Pharmacy in Košice, Slovakia, for analysis. Within 4 h of collection, all samples were analyzed microbiologically.

The samples were diluted decimally according to ISO 6887-5 [19]. Then, 1.0 ml of each diluted sample was placed into sterile Petri dishes, poured with Violet Red Bile Glucose Agar (Merck, Germany) as a selective agar for *Enterobacteriaceae*, and incubated at 37°C for 24 h. After incubation, the bacterial colonies were counted on each Petri dish. Each milk sample was evaluated by determining the number of colony-forming units (CFU) in 1 ml and then recalculated to log_10_. Based on their characteristic appearance, five typical colonies per plate were selected, purified on Brain Heart Infusion Agar (Merck, Germany), and stored at –18°C for further investigation.

The DNA was extracted from the isolates according to Hein et al. [20]. PCR was used to identify the *Enterobacteriaceae* family according to Ke et al. [21]. In our study, the term *“Enterobacteriaceae”* refers to taxa classified within the family *Enterobacteriaceae* according to the post-2016 taxonomic revision. The *16S rRNA* gene sequences marked as Ent-F 5′-CGT TAC YCG CAG AAG AAG CA-3′ and Ent-R 5′-CTG AGC GTC AGT CTT YGT CC-3′, which covered the identification of 89% of the genus of the family *Enterobacteriaceae* (sensu stricto) [2], were used. Due to possible overlap of genera that no longer belong to the *Enterobacteriaceae* family, all collected colonies were identified by PCR and MALDI-TOF MS. Each primer was synthesized in Metabion (Germany), and the size of the product was 259 bp. The PCR protocol was optimized as follows: initial denaturation at 95°C for 13 min, followed by 30 cycles (denaturation at 95°C for 20 sec, annealing at 52°C for 30 sec, and extension at 72°C for 2 min). The final extension was performed at 72°C for 10 min. The HotFirepol^®^ Mastermix (Solis BioDyne, Tart, Estonia) was used in the PCR. The PCR products were visualized on a 1.5% agarose gel stained with Goldview^TM^ nucleic acid stain (Beijing SBS Genetech Co., Ltd., Beijing).

All isolates examined were streaked on Columbia Blood Agar (Merck, Darmstadt, Germany) to prepare for MALDI-TOF MS examination and incubated at 37°C for 24 h. Individual samples were prepared via an extraction procedure using ethanol and formic acid [22]. Then, the colonies were used for identification by MALDI-TOF MS (Bruker Daltonics, Billerica, MA, USA).

### 2.3. Antimicrobial susceptibility testing

The identified isolates of the family *Enterobacteriaceae* were tested for antibiotic susceptibility using the disc diffusion method (DDM) according to the procedure described in the CLSI document [23]. Ten antibiotics were tested: ampicillin (AMP, 10 µg), cefoxitin (FOX, 30 µg), ceftizoxime (ZOX, 30 µg), cefazolin (CZ, 30 µg), kanamycin (K, 30 µg), streptomycin (S, 10 µg), tetracycline (TE, 30 µg), gentamicin (CN, 10 µg), ofloxacin (OFX, 5 µg), and chloramphenicol (C, 30 µg).

Antibiotics were selected based on the most commonly used antimicrobial agents in veterinary and human medicine in Slovakia for treatment [24, 25]. *Escherichia coli* ATCC 25922 was used as a reference strain. The results were evaluated according to two systems, namely the European Committee on Antimicrobial Susceptibility Testing [26] and the Clinical and Laboratory Standards Institute guidelines [23]. According to the CLSI system, it was possible to evaluate all ten tested antibiotics, and according to the EUCAST system, it was possible to assess susceptibility to these antibiotics, namely ampicillin (AMP, 10 µg), cefoxitin (FOX, 30 µg), ofloxacin (OFX, 5 µg), gentamicin (CN, 10 µg), and chloramphenicol (C, 30 µg). The discs used for susceptibility testing were manufactured by HIMEDIA (Mumbai, India). The diameters of the inhibition zones were recorded in millimeters (mm) and interpreted as susceptible, intermediate-susceptible, or resistant.

### 2.4. Detection of antimicrobial-resistant genes

*Enterobacteriaceae* isolates for the resistance genes *bla*_TEM_ (Figure 6A), *ampC* (Figure 6B), and *bla*_CTX–M_ (Figure 7), were tested. Genes that can confer AMR to beta-lactam antibiotics were detected in PCR using the gene-specific primers shown in Table 1 [27, 28]. The PCR protocol was optimized as follows: initial denaturation at 95°C for 13 min, followed by 30 cycles of denaturation at 95°C for 20 sec, annealing at different temperatures depending on the gene for 30 sec, and extension at 72°C for 2 min. The final extension was performed at 72°C for 10 min. PCR amplicons were run on 1.5% agarose gel. The expected sizes of the PCR products varied among genes (Table 1).

**Table 1.** The primers used in this study for the detection of resistance genes in *Enterobacteriaceae* isolates using a PCR-based method.

Gene	Primer sequence (5′-3′)	Product size (bp)	Annealing temperature	References
*bla*_TEM_	TCG CCG CAT ACA CTA TTC TCA GAA TGA	445	61°C	[27]
	ACG CTC ACC GGC TCC AGA TTT AT			
*ampC*	CGC CTC TTG CTC CAC AT	491	51°C	[28]
	CGC CGA ACA AAC CGA TA			
*bla*_CTX–M_	ATG TGC AGY ACC AGT AAR GTK ATG GC	593	65°C	[27]
	TGG GTR AAR TAR GTS ACC AGA AYC AGC GG			

bp, basic pairs.

### 2.5. Sequencing

In isolates with identified genes *bla*_TEM_, *bla*_CTX–M_, and *ampC*, the corresponding products of the specified size were sequenced using the Sanger method by SEQme s.r.o. (Dobříš, Czech Republic). The sequences obtained were analyzed for homology with the sequences available in the GenBank-EMBL database using the BLAST program (NCBI, software package 3.40, Bethesda, MD, USA).

### 2.6. Data analysis

Data were entered, cleaned, and validated in a Microsoft^TM^ Excel spreadsheet (MS Office Excel^®^ 2021, Wanchai, Hong Kong). The average number of family *Enterobacteriaceae* in the milk samples was recalculated to log_10_ transformation. The distribution of each species in the milk samples was determined by calculating the percentage of each species out of the total number of isolated bacteria. The PCR results (positive or negative) were reference variables for descriptive analyses. Univariate analyses were conducted for descriptive statistics, and the data was presented as percentages. Statistical methods of basic statistics such as arithmetic mean, sample standard deviation, median, coefficient of variation, minimum, and maximum were used in the work to evaluate the counts of *Enterobacteriaceae* in milk samples.

## 3. Results

### 3.1. Isolation and enumeration of the family Enterobacteriaceae

*Enterobacteriaceae* were present in 12 (85.7%) of the 14 raw bulk milk samples tested. The average count in raw cow’s milk samples was 3.27 log_10_ ± 1.94 CFU/ml (colony-forming units, CFU). For the positive raw milk samples, the average count was 3.81 log_10_ ± 1.47 CFU/ml (Table 2). In all milk samples tested after batch pasteurization, no *Enterobacteriaceae* were detected. All dairy plants where the samples were taken use, in accordance with current legislation, a combination of low temperature and a longer pasteurization time to protect the milk proteins before cheesemaking.

**Figure 1.**
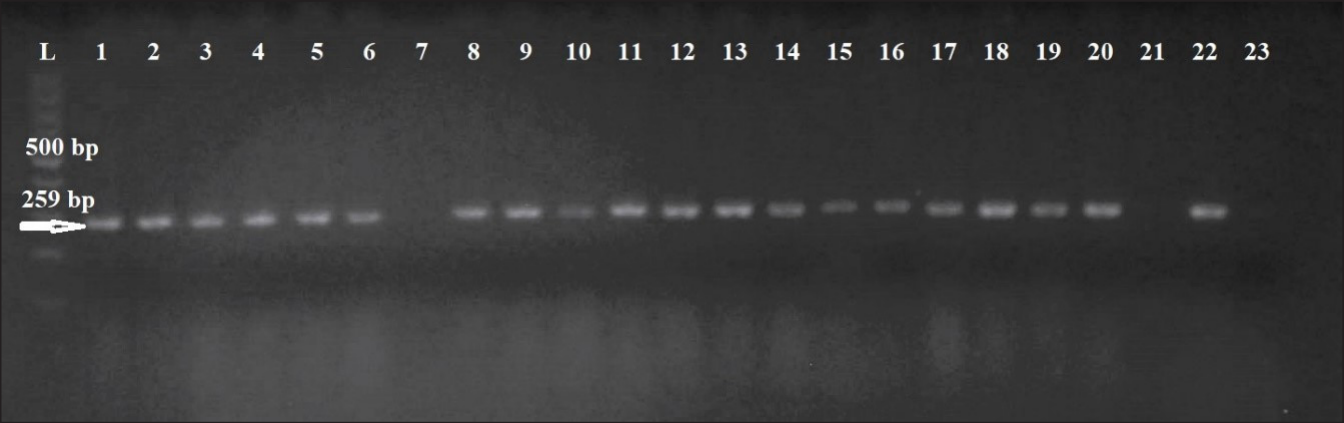
Agarose gel electrophoresis of the PCR product of *16S rRNA* gene sequences of the family *Enterobacteriaceae*. Lane L: size marker; Lanes 1–6, 8–20: positive samples; Lanes 7, 21: negative samples; Lane 22: positive control; Lane 23: negative control.

53 of 59 isolates examined were positive after PCR (Figure 1) using primers that should cover 89% of the genus within the family *Enterobacteriaceae* [2]. All 59 isolates were simultaneously identified using MALDI-TOF MS. Isolates confirmed by MALDI-TOF MS to belong to genera that were originally part of *Enterobacteriaceae* but have since been reassigned to other families within the order *Enterobacterales* were excluded from further analysis. The use of this primer set identified all 53 isolates belonging to the *Enterobacteriaceae* family at the family level, but genera currently outside this family were also identified as positive. Therefore, MALDI-TOF MS identification was appropriate, unambiguously determining that the isolates no longer belong to *Enterobacteriaceae*. Only isolates with a MALDI-TOF MS score higher than 2.299, indicating high confidence at the species level, were included. Finally, identification by MALDI-TOF MS showed that all 59 isolates belonged to the order *Enterobacterales* (six isolates had negative PCR results), but only 30 isolates belonged to the *Enterobacteriaceae* family according to current taxonomy. The complete dataset is available in Supplementary Table S1. In general, the most common genus was *Hafnia*, belonging to the *Hafniaceae* family, identified by MALDI-TOF MS. Other isolates confirmed by PCR, but outside the *Enterobacteriaceae*, were *Serratia liquefaciens* and *Pantoea agglomerans*, which belong to the family *Yersiniacae* and family *Erwiniaceae*, order *Enterobacterales*.

**Table 2.** Enumeration of *Enterobacteriaceae* in milk samples from artisanal dairy plants in Slovakia.

	**Total samples (raw milk)**	**Positive samples (raw milk)**	**Pasteurized milk**
Average in log_10_	3.27 CFU/ml	3.81 CFU/ml	0.0 CFU/ml
Standard deviation	± 1.94	± 1.47	± 0.0
Median in log_10_	3.38 CFU/ml	3.90 CFU/ml	0.0 CFU/ml
Coefficient of variation	0.59	0.39	0.0
Minimum in log_10_	0.00 CFU/ml	1.3 CFU/ml	0.0 CFU/ml
Maximum in log_10_	6.24 CFU/ml	6.24 CFU/ml	0.0 CFU/ml
Positive samples (number)	12 (85.7%)	–	0
Total samples (number)	14	–	14

**Figure 2.**
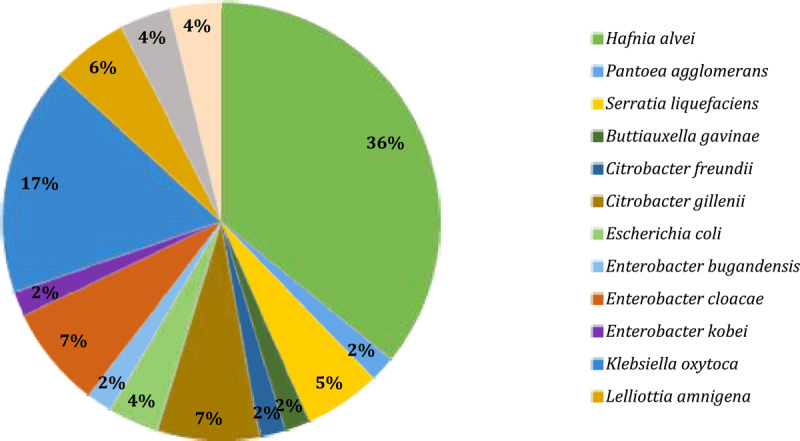
MALDI-TOF identification result: A total of 53 confirmed isolates belonging to the order *Enterobacterales* (including family *Hafniaceae, Yersiniaceae, Erwiniaceae*) of which 30 isolates are part of the family *Enterobacteriaceae*.

**Figure 3.**
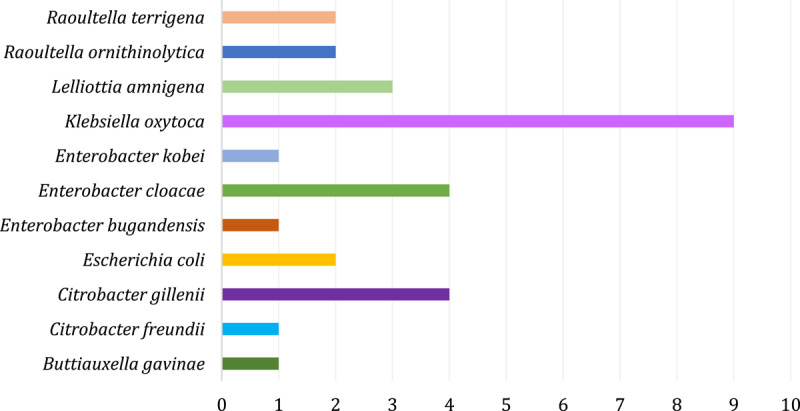
MALDI-TOF identification result: Numerical representation of individual species of the *Enterobacteriaceae* family (30) obtained after their isolation from samples of raw cow’s milk with the highest incidence of *Klebsiella oxytoca* (30.0%).

In Figure 2, a result is obtained after identification at the species level by MALDI-TOF MS. Finally, 30 of 53 isolates belonged to the family *Enterobacteriaceae*. In the samples taken (Figure 3), experimental analysis confirmed the presence of eleven species of the family *Enterobacteriaceae*. Overall, the most common species was *Klebsiella oxytoca* (9, 30.0%), followed by *Enterobacter cloacae* and *Citrobacter gillenii* (4, 13.3%). In our study, *Enterobacteriaceae* refers to the family as amended in 2016 (order *Enterobacterales*) and includes only genera still within this family.

### 3.2. Antimicrobial susceptibility test results for representatives of family Enterobacteriaceae

The antimicrobial susceptibility results were evaluated separately according to the CLSI 2023 and EUCAST 2023 documents due to the expectation differences between these two systems. Evaluation using the CLSI criteria was possible for all selected antibiotics, and evaluation using the EUCAST criteria for the *Enterobacteriaceae* family was possible for five antibiotics (FOX, AMP, OFX, CN, C). The zone diameter breaking points defined by CLSI are different compared to the values defined by EUCAST. The results according to CLSI and EUCAST for all isolates tested from milk samples are presented in Table 3.

**Table 3.** Antimicrobial-resistant phenotypes of *Enterobacteriaceae* identified by MALDI-TOF MS according to CLSI and EUCAST.

Resistant										
	ZOX	FOX	AMP	K	CZ	OFX	S	CN	C	TE
CLSI	12 (40.0%)	22 (73.3%)	19 (63.3%)	1 (46.7%)	14 (46.7%)	1 (3.3%)	12 (40.0%)	5 (16.7%)	0 (0.0%)	3 (10.0%)
EUCAST	Nda	26 (86.7%)	19 (63.3%)	Nda	Nda	16 (53.3%)	Nda	17 (56.7%)	0 (0.0%)	Nda

Nda, no data available; ZOX, ceftizoxime; FOX, cefoxitin; AMP, ampicillin; K, kanamycin; CZ, cefazolin; OFX, ofloxacin; S, streptomycin; CN, gentamicin; C, chloramphenicol; TE, tetracycline.

**Figure 4.**
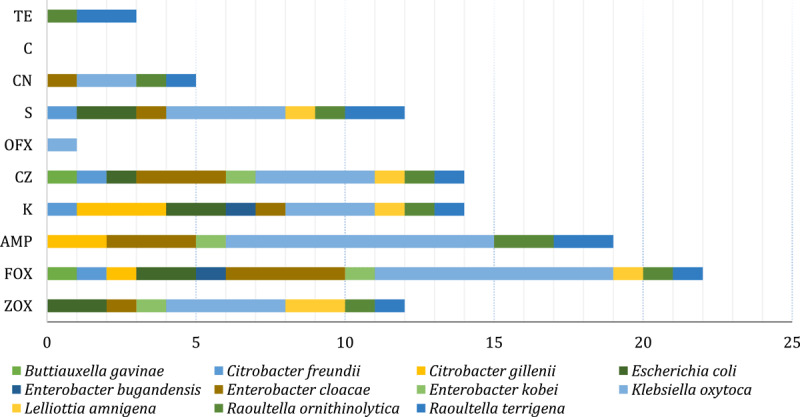
Distribution of antimicrobial-resistant phenotypes of *Enterobacteriaceae* identified by MALDI-TOF MS at species level according to the CLSI system. ceftizoxime (ZOX), cefoxitin (FOX), ampicillin (AMP), kanamycin (K), CZ (cefazolin), ofloxacin (OFX), streptomycin (S), gentamicin (CN), chloramphenicol (C), tetracycline (TE).

Although numerically prevalence differs, in many cases the trend was similar (e.g., cefoxitin and ampicillin are top resistance in both systems, just at different absolute percentages). As already mentioned, it was possible to compare five antibiotics with each other. In particular, the number of resistant isolates was lower under the CLSI criteria than under the EUCAST criteria for the same antibiotics. The most significant differences were observed with ofloxacin and gentamicin; only one isolate was evaluated as resistant to ofloxacin by the CLSI, versus 16 by the EUCAST system. In all isolates investigated, the highest resistance to cephalosporins was observed, according to EUCAST to cefoxitin in 26 (86.7%) isolates and according to CLSI to cefoxitin in 22 (73.3%) followed by ampicillin in 19 (63.3%) isolates according to both systems. A high level of resistance was observed in isolates against gentamicin (56.7%) and ofloxacin (53.3%), but only according to the EUCAST criteria. The number and distribution of antimicrobial-resistant phenotypes of *Enterobacteriaceae* by species according to CLSI (Figure 4) showed that all nine isolates (100%) of *K. oxytoca* were resistant to ampicillin and eight (88.9%) of these isolates were resistant to cefoxitin. All five isolates of the genus *Enterobacter* were resistant to cefoxitin. Resistance to both isolates of *Raoultella terrigena* was observed simultaneously against ampicillin, streptomycin, and tetracycline. According to these criteria, only one isolation of *K. oxytoca* was resistant to ofloxacin. No resistance was observed in one isolation of *Lelliottia amnigena* to all antibiotics tested.

**Figure 5.**
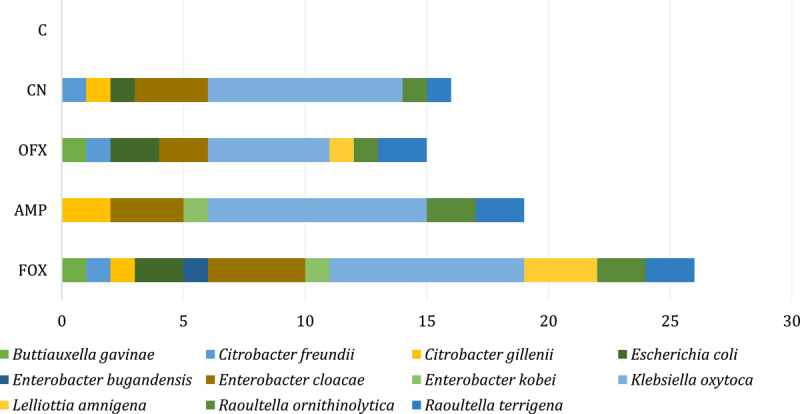
Distribution of antimicrobial-resistant phenotypes of *Enterobacteriaceae* identified by MALDI-TOF MS at species level according to the EUCAST system. cefoxitin (FOX), ampicillin (AMP), ofloxacin (OFX), gentamicin (CN), chloramphenicol (C).

The number and distribution of antimicrobial-resistant phenotypes of *Enterobacteriaceae* by species according to the EUCAST system are illustrated in Figure 5. This system could evaluate five antibiotics and showed resistance in all nine (100%) *K. oxytoca* to cefoxitin, the same result as in the case of the CLSI criteria. On the other hand, the most significant differences from CLSI were found in the assessment of resistance to ofloxacin and gentamicin, regarding the high number of resistant isolates and the greater variability at the species level.

### 3.3. Detection of resistance genes among all isolates of family Enterobacteriaceae

The assessment of *Enterobacteriaceae* safety through *in vitro* expression of virulence traits does not always reflect the real hazard in these groups of bacteria due to the presence of silent genes. Bacteria in raw milk can be under antibiotic pressure during the treatment of animals with mastitis, and could potentially be activated by environmental conditions, thus improving the pathogenicity of these bacteria [16]. In our study, the presence of resistance genes (*bla*_TEM_, *bla*_CTX–M_, *ampC*) was investigated in all isolates with or without phenotypic expression.

The phenotypic expression of resistance against ceftizoxime, cefoxitin, cefoxitin, ampicillin according to both systems and the presence of resistance genes in the identified isolates are represented in Table 4. As documented in the table, among all isolates identified belonging to the *Enterobacteriaceae* family, there were 20 (66.7%) isolates with confirmed *bla*_TEM_. Most of them were identified as *K. oxytoca*. Overall, the resistance gene *ampC* was found in eleven isolates, including seven species of the family. The primers used are designed to detect plasmid *ampC* resistance genes [28] and do not amplify the innately present chromosomal *ampC* genes in the several species of *Enterobacteriaceae*. Nine isolates identified as *K. oxytoca* and one of *C. gillenii* harbored the *bla*_CTX-M_ gene. As shown in Table 4, there are differences in resistance phenotype expression against cefoxitin (FOX1, FOX2) only in *Lelliottia* spp. and *Raoultella* spp.

**Table 4.** Antimicrobial-resistant phenotypes of *Enterobacteriaceae* and number of isolates with confirmed resistance genes *bla*_TEM_, *bla*_CTX–M_, and *ampC*.

Isolates	Antibiotics					Genes			Number of genes		
**ZOX**	**FOX1**	**FOX2**	**AMP**	**CZ**	***bla*_TEM_**	***ampC***	***bla*_CTX–M_**	**0**	**1**	**2**
*Buttiauxella gavinae*	–	1	1	–	1	–	–	–	1	–	–
*Citrobacter freundii*	–	1	1	–	1	1	1	–	–	–	1
*Citrobacter gillenii*	–	1	1	2	–	3	–	1	1	2	1
*Escherichia coli*	2	2	2	–	1	1	2	–	–	1	1
*Enterobacter bugandensis*	–	1	1	–	–	–	1	–	–	1	–
*Enterobacter cloacae*	1	4	4	3	3	4	3	–	–	1	3
*Enterobacter kobei*	1	1	1	1	1	–	1	–	–	1	–
*Klebsiella oxytoca*	4	8	8	9	4	6	–	9	–	3	6
*Lelliottia amnigena*	2	1	3	–	1	1	–	–	2	1	–
*Raoultella ornithinolytica*	1	1	2	2	1	2	1	–	–	1	1
*Raoultella terrigena*	1	1	2	2	1	2	2	–	–	–	2
Total	12	22	26	19	14	20	11	10	4	11	15

ceftizoxime (ZOX–CLSI), cefoxitin (FOX1–CLSI), cefoxitin (FOX2–EUCAST), ampicillin (AMP–CLSI, EUCAST), cefazolin (CZ–CLSI).

**Figure 6.**
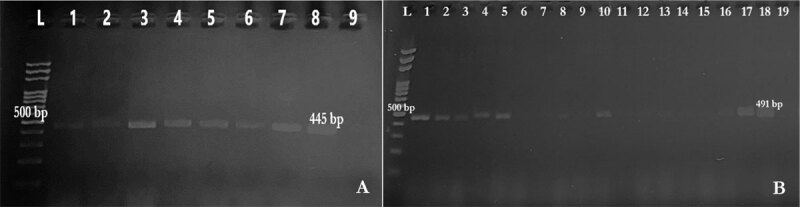
A. Agarose gel electrophoresis of PCR product of *bla*_TEM_ (445 bp). Lane L: size marker; Lanes 1–7: positive samples; Lane 8: positive control; Lane 9: negative control (A). B. Agarose gel electrophoresis of PCR product of *ampC* (491 bp). Lane L: size marker; Lanes 1–5, 10, 17: positive samples; Lanes 6 – 9, 11–16: negative samples, Lane 18: positive control; Lane 19: negative control (B).

**Figure 7.**
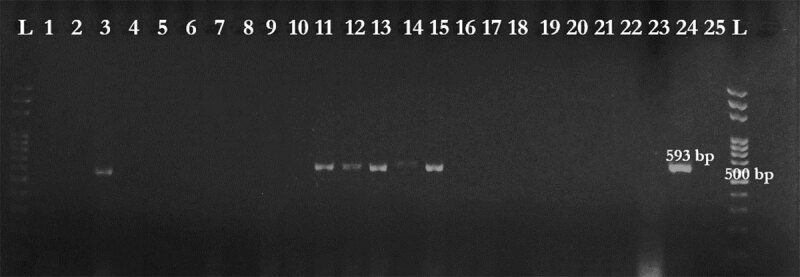
Agarose gel electrophoresis of PCR product of *bla*_CTX-M_; Lane L: size marker; Lanes 3, 11–15: positive samples; Lanes 1, 2, 4–10, 16–23 negative samples; Lane 24: positive control; Lane 25: negative control.

In general, in 26 isolates identified from the *Enterobacteriaceae* family, one or two genes were confirmed. Half of all identified isolates had two genes simultaneously. In six isolates confirmed as *Klebsiella oxytoca*, a combination of the *bla*_TEM_ and *ampC genes* was found. Four isolates, namely *Buttiauxella gavinae* (1), *Citrobacter gillenii* (1), and *Lelliottia amnigena* (2), did not have the presence of *bla*_TEM_/*bla*_CTX-M_/*ampC*.

## 4. Discussion

Family *Enterobacteriaceae* are a group of bacteria that serve as indicators of milk collection hygiene at the farm level. They are also indicators of poor hygiene in milk processing operations at dairies, and consequently, the *Enterobacteriaceae* can also be detected in final dairy products.

In our study, the presence of *Enterobacteriaceae* was detected in 12 (85.7%) samples of raw cow’s milk, with an average count of 3.81 log_10_ ± 1.47 CFU/ml. The number of positive samples ranged from 1.3 to 6.24 log_10_ CFU/ml. An important finding regarding the effectiveness of heat treatment in dairy plants was that no *Enterobacteriaceae* were detected after pasteurization, indicating an effective process, but also suggesting that the main findings of our study on resistance could apply only to raw milk bacteria.

In the study by Farhat et al. [29], the presence of *Enterobacteriaceae* was investigated using selective VRBG agar, and *Enterobacteriaceae* were detected in all 50 (100%) raw milk samples. The average count reported in their study was significantly higher than our findings, at 7.41 log_10_ CFU/ml. In another work by Tepeli and Zorba [30], the total number of Enterobacteriaceae ranged from 3.0 to 5.0 log_10_ CFU/ml in farm milk and from 4.0 to 7.0 log_10_ CFU/ml in pooled milk samples. A study conducted in Egypt analyzed 100 raw milk samples and found *Enterobacteriaceae* in 84 samples, with an average of 6.00 log_10_ ± 5.30 CFU/ml. The highest frequency of positive samples (48.81%) fell within the range of 5.0 to 6.0 log_10_ CFU/ml [31].

As mentioned in the results section, the most common species detected in our work was *Klebsiella oxytoca*, followed by *Enterobacter cloacae* and *Citrobacter gillenii*. Their presence in bulk tank milk could be due to subclinical mastitis in cows. However, this assertion is limited by the lack of information on the animals’ health status and the antimicrobial use practices on the farms.

A study by Slovak authors published in 2017 reported that enterobacteria in raw milk samples collected in Slovakia ranged from 0.00 to 4.34 log_10_ CFU/ml [32]. Compared with our study, the maximum *Enterobacteriaceae* count in that study was lower.

Another study in Slovakia examined the representation of individual bacterial species within the family *Enterobacteriaceae*, in which the majority of isolates belonged to *Citrobacter* spp. (21.4%), followed by isolates of *Shigella* spp. (20.0%), *E*. coli (14.2%) and *Enterobacter* spp. (12.8%) [33]. The identification of individual bacterial species in this study was performed similarly to our method using MALDI-TOF MS. Similar results were also presented in the work of Kagkli et al. [34], where the most common genus of the family *Enterobacteriaceae* in raw milk was *Escherichia, Enterobacter, Klebsiella*, and *Citrobacter*, which was consistent with the results of our research. A 2020 study in Egypt also investigated the presence of *Enterobacteriaceae* in raw milk. This study also included genera that have already been assigned to separate families under the new taxonomic classification. Representatives of the *Hafniaceae* (17.65%) and *Yersiniaceae* (*Serratia* spp., 25.81%) families were among the most frequently isolated from raw milk in this study. Both families currently belong to separate orders within the *Enterobacterales*. Among the genera of the family *Enterobacteriaceae, Klebsiella* (15.69%), *Enterobacter* (7.84%), *Escherichia* (6.54%), and *Citrobacter* (3.92%) were recorded, constituting a minority [31].

The situation regarding AMR shows specificities depending on therapeutic practices in human and veterinary medicine in a given country. This applies to both human and animal populations and their mutual connection. Hleba et al. [35] investigated the susceptibility of *Enterobacteriaceae* to selected antibiotics in milk collected on farms in Slovakia. In this work, antibiotic susceptibility was assessed according to EUCAST criteria. The highest resistance to ampicillin was recorded in milk (57.14%). Compared to our results, these values were comparable. Resistance to streptomycin was 14.28% among milk isolates; according to current EUCAST criteria, no established values are available for this antibiotic. It is interesting to compare resistance to gentamicin: in 2011, it was not detected in raw milk isolates; in contrast, in our study, resistance was detected in milk isolates at almost 60%. It is necessary to account for the significant time gap between the works. The same Slovak authors published another work in 2015, whose objective was to investigate AMR in *Enterobacteriaceae* isolates from milk and dairy products, and they found results similar to those of their previous work with ampicillin. Similarly, four years later, they did not record resistance to gentamicin. For this reason, it is very important to evaluate AMR in the given country and its antibiotic treatment practices. Intrinsic resistance of some *Enterobacteriaceae* to beta-lactam antibiotics is due to the presence of inducible chromosomal *ampC* beta-lactamases, low outer membrane permeability, and active efflux mechanisms. These aspects need to be considered, especially when evaluating the phenotypic manifestation of beta-lactam antibiotic resistance [36].

Raw milk is also a reservoir of antimicrobial resistance genes [36, 37]. In a review of the AMR situation over the past 10 years, which examined 306 studies, it was noted that when investigating resistance and resistance genes in milk, the greatest attention is generally focused on the genus *Staphylococcus*, and the samples collected primarily represent milk from mastitis dairy cows [38].

Many studies investigating the presence of ESBL genes in *Enterobacteriaceae* first select isolates based on ESBL phenotype. In a 2024 study [39], the presence of *bla*_TEM_, *bla*_SHV_, and *bla*_CTX-M_ resistance genes were investigated in isolates from milk and swab samples collected from the milk collection environment in Ethiopia. The dominant isolates were those in which the presence of the *bla*_TEM_ (85.0%) and *bla*_CTX–M_ (78.8%) genes were confirmed. This representation of isolates with confirmed resistance genes was significantly higher than in our work. However, it is necessary to add, for the objectivity of the comparison of the results, that in the study from Ethiopia, resistance genes were investigated only in the so-called ESBL producers. Dey et al. [40] analyzed ESBL-, MBL-, and *ampC*-producing *Enterobacteriaceae* in raw milk from farms and dairies. In addition to other ESBL genes, *bla*_TEM_ and *bla*_CTX–M1_ were also examined in ESBL producers. In this work, ESBL genes were detected in 9 isolates, representing 2.13% of all *Enterobacteriaceae* examined. In our work, there were 15 isolates with detected *bla*_TEM_ and *bla*_CTX–M_ genes in raw milk samples, which had a representation of these genes individually or in combination. This study also examined the presence of the *ampC* genes in milk, which was detected in 28 isolates (6.65%) of the total number, which could not be compared with our data, since the mentioned study investigated several genes responsible for *ampC* production. In our work, some isolates’ beta-lactam resistance was likely due to intrinsic chromosomal enzymes that were not detected by the chosen primers. It should be noted that our study did not include several other clinically relevant beta-lactamases. For example, *K. oxytoca* produces the intrinsic OXY beta-lactamase, and in some studies, the presence of *Klebsiella* spp. has been investigated. *bla*_SHV_, *bla*_IMP_, *bla*_OXA_ genes [38, 40]. In our work, the selected PCR targets (*bla*_TEM_, *bla*_CTX–M_, and plasmid-mediated *amp*C) cover the major ESBL and *ampC* plasmid families, but they do not encompass all possible beta-lactamase genes. This represents a limitation, as additional resistance determinants may have remained undetected.

However, our expectation of detecting silent genes in phenotypically susceptible isolates was not confirmed. This suggests that *Enterobacteriaceae* in raw milk may be selected by prior antimicrobial exposure. As mentioned, the limitation of sample size can have lacked any silent genes, and it is essential to consider the intrinsic resistance of some species of *Enterobacteriaceae* against some beta-lactam agents. From this perspective, awareness of AMR development at the individual farm level is essential for managing bacterial mastitis and other infections.

## 5. Conclusions

This study investigated the presence of the family *Enterobacteriaceae* (sensu stricto) as a potential source of AMR in milk before and after pasteurization. *Enterobacteriaceae* were present in 12 of all the milk samples tested (raw and pasteurized). The relatively small sample size substantially limits our findings and limits the extent to which the results can be generalized to broader populations. Our effort was to precisely identify isolates of the family *Enterobacteriaceae* according to current taxonomy and to evaluate phenotypic resistance as objectively as possible, using two independent assessment systems. Overall, the most commonly identified species were *Klebsiella oxytoca*, followed by *Citrobacter gillenii* and *Enterobacter cloacae*. Most isolates showed resistance to cefoxitin and ampicillin. Antimicrobial susceptibility testing was performed using two systems to assess potential discrepancies. Significant differences were observed, particularly between ofloxacin and gentamicin. Resistance to ofloxacin was 53.3% according to EUCAST (2023), compared to only 3.3% based on CLSI (2023). Similarly, resistance to gentamicin was 56.7% according to EUCAST, versus 16.7% according to CLSI. Such discrepancies can significantly affect the final interpretation of resistance data and raise concerns regarding the simultaneous use of both systems in scientific studies. Importantly, no *Enterobacteriaceae* isolates were detected in any of the pasteurized milk samples. The evaluation of the efficiency of heat treatment in these dairy plants is important for public health, given our results (the presence of Enterobacteriaceae with a high prevalence of beta-lactamase genes in raw milk). Ensuring food safety must be a daily priority. Raw milk serves as the primary input for dairy products, and its microbial quality is vital for food safety. Increased awareness of bacteria that pose a health risk and the potential spread of AMR should be clear evidence to discourage the consumption of raw milk or its products, despite the long-standing tradition in some countries. Our findings highlight that raw milk can serve as a reservoir for multidrug-resistant *Enterobacteriaceae* with the potential to be transmitted to humans. Implementing strict pasteurization and hygiene measures is therefore essential to reduce this risk.

## Figures and Tables

**Supplementary Table S1.** List of all identified isolates by the PCR and MALDI-TOF MS method and their belonging to the family *Enterobacteriaceae*.

No. of isolate	PCR	MALDI-TOF MS	Family *Enterobacteriaceae*	No. of isolate	PCR	MALDI-TOF MS	Family *Enterobacteriaceae*
1	+	*Klebsiella oxytoca*	+	31	+	*Hafnia alvei*	–
2	+	*Klebsiella oxytoca*	+	32	+	*Enterobacter cloacae*	+
3	+	*Lelliottia amnigena*	+	33	+	*Hafnia alvei*	–
4	+	*Hafnia alvei*	–	34	+	*Hafnia alvei*	–
5	+	*Lelliottia amnigena*	+	35	+	*Klebsiella oxytoca*	+
6	+	*Hafnia alvei*	–	36	+	*Serratia liquefaciens*	–
7	+	*Klebsiella oxytoca*	+	37	+	*Hafnia alvei*	–
8	+	*Lelliottia amnigena*	+	38	+	*Hafnia alvei*	–
9	+	*Citrobacter gillenii*	+	39	+	*Hafnia alvei*	–
10	+	*Hafnia alvei*	–	40	+	*Enterobacter cloacae*	+
11	+	*Enterobacter cloacae*	+	41	+	*Hafnia alvei*	–
12	+	*Hafnia alvei*	–	42	+	*Klebsiella oxytoca*	+
13	+	*Hafnia alvei*	–	43	+	*Enterobacter cloacae*	+
14	+	*Hafnia alvei*	–	44	–	*Hafnia alvei*	–
15	–	*Hafnia alvei*	–	45	+	*Hafnia alvei*	–
16	–	*Hafnia alvei*	–	46	+	*Citrobacter gillenii*	+
17	+	*Klebsiella oxytoca*	+	47	+	*Klebsiella oxytoca*	+
18	+	*Hafnia alvei*	–	48	–	*Hafnia alvei*	–
19	+	*Citrobacter gillenii*	+	49	+	*Serratia liquefaciens*	–
20	+	*Buttiauxella gavinae*	+	50	+	*Enterobacter kobei*	+
21	+	*Hafnia alvei*	–	51	–	*Hafnia alvei*	–
22	+	*Serratia liquefaciens*	–	52	–	*Hafnia alvei*	–
23	+	*Citrobacter gillenii*	+	53	+	*Escherichia coli*	+
24	+	*Klebsiella oxytoca*	+	54	+	*Raoultella terrigena*	+
25	+	*Hafnia alvei*	–	55	+	*Klebsiella oxytoca*	+
26	+	*Raoultella ornithinolytica*	+	56	+	*Raoultella ornithinolytica*	+
27	+	*Hafnia alvei*	–	57	+	*Escherichia coli*	+
28	+	*Pantoea agglomerans*	–	58	+	*Raoultella terrigena*	+
29	+	*Hafnia alvei*	–	59	+	*Citrobacter freundii*	+
30	+	*Enterobacter bugandensis*	+				

## Data Availability

The data presented in this study are available from the corresponding author upon reasonable request.

## List of abbreviations

AMP, ampicillin; ampC, aminopenicillins inactivated by cephalosporinases; AMR, antimicrobial resistance; BLAST, Basic Local Alignment Search Tool; C, chloramphenicol; CFU, colony forming units; CLSI, Clinical and Laboratory Standards Institute; CN, gentamicin; CP, carbapenemases; CZ, cefazolin; DDM, disc diffusion method; DNA, Deoxyribonucleic Acid; EMBL, European Molecular Biology Laboratory; ESBL, extended-spectrum beta-lactamase; EUCAST, European Committee on Antimicrobial Susceptibility Testing; FOX, cefoxitin; GMP, Good Manufacturing Practice; HACCP, Hazard Analysis and Critical Control Point; ISO, International Organization for Standardization; K, kanamycin; MALDI-TOF MS, Matrix-Assisted Laser Desorption/Ionization Time-of-Flight Mass Spectrometry; MBL, metallo-beta-lactamases; OFX, ofloxacin; PCR, polymerase chain reaction; pH, potential of hydrogen, S, streptomycin; *16S rRNA*, 16S ribosomal ribonucleic acid; TE, tetracycline; VRBG, violet red bile glucose; ZOX, ceftizoxime; °C, degree Celsius; gm, gram; h, hour; µg, microgram; ml, milliliter; mm, millimeter; min, minute; sec, second.
